# Multidisciplinary medication review during older patient hospitalization according to STOPP/START criteria reduces potentially inappropriate prescriptions: MoPIM cohort study

**DOI:** 10.1186/s12877-024-05185-w

**Published:** 2024-07-08

**Authors:** Sara Ortonobes, Susana Herranz, Marina Lleal, Daniel Sevilla-Sánchez, Rosa Jordana, Oscar Mascaró, Olivia Ferrández, Elisabet de Jaime, Rafael Estrada, Gloria Julia Nazco, Marisa Baré, the MoPIM Study Group, Celia Corral-Vazquez, Pere Roura-Poch, Núria Solà, Javier González, Núria Molist, Mariona Espaulella, Maria Sala, Miguel Ángel Márquez, Marta Arellano, Carlos Clemente, Olga Sabartés, Núria Carballo, Marta de Antonio, Maria Olatz Ibarra, Candelaria Martin, Rubén Hernández

**Affiliations:** 1 Pharmacy Department, Parc Taulí Hospital Universitari, Institut d’Investigació i Innovació Parc Taulí (I3PT-CERCA), Universitat Autònoma de Barcelona, 08208 Sabadell, Spain; 2 Acute Care Geriatric Unit, Parc Taulí Hospital Universitari, Institut d’Investigació i Innovació Parc Taulí (I3PT-CERCA), Universitat Autònoma de Barcelona, 08208 Sabadell, Catalonia Spain; 3 Research Network On Health Services in Chronic Patients (REDISSEC), Instituto de Salud Carlos III, 28029 Madrid, Spain; 4 Clinical Epidemiology and Cancer Screening Department, Parc Taulí Hospital Universitari, Institut d’Investigació I Innovació Parc Taulí (I3PT-CERCA), Universitat Autònoma de Barcelona, 08208 Sabadell, Spain; 5 Department of Paediatrics, Obstetrics and Gynaecology, Preventive Medicine and Public Health, Autonomous University of Barcelona (UAB), 08193 Bellaterra, Spain; 6 Research Network on Chronicity, Primary Care and Health Promotion (RICAPPS), Instituto de Salud Carlos III, 28029 Madrid, Spain; 7 Pharmacy Department, Parc Sanitari Pere Virgili, 08023 Barcelona, Spain; 8 Internal Medicine Department, Parc Taulí Hospital Universitari, Institut d’Investigació i Innovació Parc Taulí (I3PT-CERCA), Universitat Autònoma de Barcelona, 08208 Sabadell, Spain; 9 Internal Medicine Department, University Hospital of Vic, Multidisciplinary Inflamation Research Group (MIRG), Facultat de Medicina, Universitat de Vic, Universitat Central de Catalunya, 08500 Vic, Spain; 10 Pharmacy Department, Consorci Parc de Salut Mar, 08003 Barcelona, Spain; 11 Geriatrics Department, Consorci Parc de Salut Mar, 08003 Barcelona, Spain; 12 Internal Medicine, Hospital Galdakao-Usansolo, 48960 Galdakao, Spain; 13 Pharmacy Department, Hospital Universitario de Canarias, 38320 La Laguna, Spain; 14 Primary Care Center, CAP Can Rull, Parc Taulí Hospital Universitari, Institut d’Investigació i Innovació Parc Taulí (I3PT-CERCA), 08206 Sabadell, Spain

**Keywords:** Potentially inappropriate prescription, Clinical committee review, Older adults, Polypharmacy, Multimorbidity, Prescription adequacy

## Abstract

**Purpose:**

Multimorbidity and polypharmacy in older adults converts the detection and adequacy of potentially inappropriate drug prescriptions (PIDP) in a healthcare priority. The objectives of this study are to describe the clinical decisions taken after the identification of PIDP by clinical pharmacists, using STOPP/START criteria, and to evaluate the degree of accomplishment of these decisions.

**Methods:**

Multicenter, prospective, non-comparative cohort study in patients aged 65 and older, hospitalized because of an exacerbation of their chronic conditions. Each possible PIDP was manually identified by the clinical pharmacist at admission and an initial decision was taken by a multidisciplinary clinical committee. At discharge, criteria were re-applied and final decisions recorded.

**Results:**

From all patients (n = 674), 493 (73.1%) presented at least one STOPP criteria at admission, significantly reduced up to 258 (38.3%) at discharge. A similar trend was observed for START criteria (36.7% *vs.* 15.7%). Regarding the top 10 most prevalent STOPP criteria, the clinical committee initially agreed to withdraw 257 (34.2%) prescriptions and to modify 93 (12.4%) prescriptions. However, the evaluation of final clinical decisions revealed that 503 (67.0%) of those STOPP criteria were ultimately amended. For the top 10 START criteria associated PIDP, the committee decided to initiate 149 (51.7%) prescriptions, while a total of 198 (68.8%) were finally introduced at discharge.

**Conclusions:**

The clinical committee, through a pharmacotherapy review, succeeded in identifying and reducing the degree of prescription inadequacy, for both STOPP and START criteria, in older patients with high degree of multimorbidity and polypharmacy.

**Trial Registration:**

NCT02830425.

**Supplementary Information:**

The online version contains supplementary material available at 10.1186/s12877-024-05185-w.

## Introduction

Progressive and constant population aging is a global phenomenon that constitutes a health challenge to societies and healthcare providers [1, 2]. Nowadays, 21.2% of people in the European Union are older than 65 years and it is predicted to exceed 30% by 2050 [3]. In parallel to this process, the multimorbidity and polypharmacy of older patients is growing, thus requiring increasing funding and human resources from public healthcare systems. In consequence, this high level of polypharmacy leads to the risk of presenting potentially inappropriate drug prescriptions (PIDP) and suffering drug-related problems [4].

PIDP have been significantly related to a variety of health-related outcomes, such as adverse drug event-related hospital admissions, functional decline and adverse drug reactions (ADR) [5]. Moreover, it is known that PIDP and ADR may exacerbate frailty features in older people, such as cognitive decline, falls or incontinence, leading to a bi-directional relationship that can result in an increased polypharmacy and higher risk of PIDP [6]. Some studies have even shown that PIDP are associated with greater risk of mortality [7, 8]. Therefore, it is essential to identify and reduce both PIDP and polypharmacy in order to optimize prescriptions in older patients. In fact, polypharmacy results in higher mortality rates in particular groups of patients, such as cognitively impaired older adults [9].

With this purpose, several explicit criteria have emerged as useful tools to identify PIDP [10]. In Europe, STOPP/START (Screening Tool of Older Person’s potentially inappropriate Prescriptions / Screening Tool to Alert doctors to Right Treatment) are the most widely used and validated criteria among older adults [11]. Its first version included 84 criteria [12] and was further enlarged in a second version including up to 114 explicit, incorporating three implicit criteria [13, 14]. These criteria were elaborated to support multidisciplinary teams in charge of multimorbid patients in the adequacy of medication associated to PIDP potentially resulting in ADR, together with the introduction of unprescribed required medication.

The application of STOPP/START criteria in several clinical settings has demonstrated its effectiveness in many different countries. In fact, these criteria have been employed to optimize medication in older patients in distinct clinical contexts, such as hospitals, primary care, nursing homes and intermediate care centers [15–17]. The use of these criteria has led to the identification of potentially inappropriate medication (PIM) and potential prescribing omissions (PPO), in direct correlation with worse health outcomes, such as emergency room visits, hospital re-admissions and mortality [18, 19].

A successful implementation of STOPP/START criteria requires a multidisciplinary team composed by physicians, nurses and clinical pharmacists, with experience in the management of geriatric patients [20]. Actually, the relevance of incorporating pharmacists in those teams has proved to be beneficial in hospital and nursing home settings, given their key role in PIM and PPO detection [21]. Moreover, medication review led by pharmacists has shown high levels of patient satisfaction towards deprescribing interventions [22]. Finally, it has been recently reported that randomized clinical trials where medication review process is led by a pharmacist, result in a reduction of polypharmacy, along with a positive impact in terms of preventing undesired hospitalizations and saving public health costs [23].

Therefore, the objectives of this study are to describe the clinical decisions taken after the identification of PIMs and PPOs by clinical pharmacists at admission of older patients, according to STOPP/START criteria and to evaluate the degree of accomplishment of these decisions at patient’s discharge, considering the evolution of patient's clinical condition. These analyses are included in MoPIM (Morbidity, Potentially Inappropriate Medication) study [24], with diverse goals related to multimorbidity, risk factors of PIDP and ADR in these patients.

## Materials and methods

### Design of the study

A multicenter and prospective non-comparative cohort study including older patients hospitalized at the internal medicine or geriatric services at five general teaching hospitals in three different regions of Spain between September 2016 and December 2018 was conducted. The detailed protocol was previously published [24].

For the purposes of this study, older patients (≥ 65 years old) admitted because of an exacerbation of their chronic pathology were included. Patients referred to home hospitalization, admitted because of an acute process not related to their chronic pathology, or with a fatal outcome expected at admission were not included. No written informed consent was deemed necessary for this study, according to the independent ethics committee.

### Data acquisition and variables

The following sociodemographic and clinical data were retrieved from the electronic health records by the clinical team responsible for the patient: patient’s code, date of birth, sex, functional status just before admission (Barthel Index), household (alone, with relatives or other people or in a nursing home), length of stay and destination at discharge from the present episode of hospitalization (home, transfer to another hospital, transfer to a nursing home). Chronic active conditions were recorded from a consensual list of 64 conditions containing all chronic diseases of the Charlson Comorbidity Index and including some risk factors as well. This index was calculated, adjusted by age and categorized by tertiles (2–5, 6–8, 9–14). Geriatric syndromes and risk factors were also recorded from a list of 15 (see published protocol [24]).

The number of chronic medications in the electronic prescription and the STOPP/START criteria [13] detected at the time of admission, with the active principle involved, were manually collected by the clinical pharmacist of each team. The second version of STOPP/START was employed and consists of 80 STOPP criteria (which detect medication that would not meet criteria for indication to a patient or a specific clinical situation or medications prescribed included drug-drug and drug-disease interactions) and 34 START criteria (which detect medication that would be recommended to incorporate, including some vaccines) [13]. The criteria are directed to prevalent diseases in older patients, are ordered by physiological systems and are easy to relate to active diagnoses.

### Clinical committee review process

A clinical committee constituted by an internal medicine or geriatrics physician, a clinical pharmacist and a nurse monitored each patient until hospital discharge (or death during hospitalization), with data collection during the initial days of admission to hospital ward and at the time of discharge. The medication review process was part of the usual patient care routine in all participating centers. Medication was only considered chronic if prescribed at least 3 months before admission, and creams, ointments, healing materials and over-the-counter medicines were not considered. Active principles were considered individually when registering STOPP/START criteria, regardless of the administered drug combinations.

For each patient, possible PIDPs were recorded at admission and, following the usual practice, the clinical committee evaluated the PIDP together with the possible actions to be taken for medication adequacy according to the STOPP/START criteria. After the committee review process, an initial clinical decision was taken for each PIDP detected. The initial decisions for PIM were classified in medication withdrawal, modification of dosage or administration frequency or medication maintenance (either with or without medical justification at admission). In the case of PPO, the initial decisions considered were modification (referring to initiation of a required medication) or maintenance of prescription omission (either with or without medical justification at admission).

For the purposes of the study, the criteria were manually re-applied by the clinical pharmacist to the prescribed medication at discharge, once acute exacerbation of clinical condition was resolved, in this case without evaluation by the clinical committee. After this process, each PIDP detected was recorded as “amended” or “maintained”, considered as final clinical decision. PIM were located to the “amended” group when medication was finally withdrawn or modified, whereas PPO were assigned to this group in case of treatment initiation before discharge.

### Sampling and analysis

All STOPP/START criteria were assessed, except for START criteria I (vaccines) due to difficulties of some centers in accessing this information. Regarding the implicit criterion STOPP A1 and given its high frequency, it was divided into the following categories according to the active principles involved: proton pump inhibitors (PPI), hypolipidemic, analgesics, acetylsalicylic acid, antihypertensives and others [25].

Data from patients initially included in the study, who died during hospitalization, were not considered for the analysis.

Binary variables were created to describe the presence of any STOPP and any START criteria at admission and at discharge and numerical variables were similarly created for the number of STOPP and number of START at admission and at discharge. For each STOPP and START criteria detected, a percentage of change between the number of patients with those criteria at admission and discharge was calculated: (N discharge – N admission) / N admission *100.

Descriptive analyses were performed for all variables. Bivariate analyses were performed to compare the presence of any STOPP at admission versus discharge (using the McNemar's Chi-squared test with continuity correction) and to compare the number of STOPP at admission versus discharge (using Wilcoxon rank sum test with continuity correction). The same analyses were performed for START criteria.

Sankey diagrams were generated with the top 10 most frequent PIM and PPO detected, illustrating the different flows from the number of PIDP detected at admission, to the initial clinical decision, and then the final decision and the amount of PIDP at discharge.

All analyses were performed with R (R Foundation for Statistical Computing, Vienna, Austria).

## Results

### Sociodemographic and clinical characteristics of the cohort

A total of 674 patients were finally included in the analysis. Median age of patients was 84.1 years (SD ± 7.0), with 52.8% of females and a mean Barthel Index of 67. According to this index, 36.6% of patients presented a dependency degree from moderate to total. In terms of household, only 12.6% lived at nursing homes before admission, while this percentage increased up to 15.6% after discharge. Almost 70% of patients were admitted for less than two weeks at the hospital setting. All remaining sociodemographic and clinical variables are summarized in Supplementary Table S1. Chronic active conditions and geriatric syndromes registered in this cohort of patients are shown in Supplementary Tables S2 and S3.

### STOPP/START criteria detection in patients’ cohort at admission and at discharge

The analysis of total prescriptions across all hospitals involved in the study revealed an average of 10.6 medications/patient at admission. This ratio was slightly increased up to 11.1 at patients’ discharge, demonstrating the high degree of polypharmacy in the studied population. From all patients, 493 (73.1%) presented at least one STOPP criteria at admission, with an average of 1.6 per patient. These numbers were significantly reduced at discharge up to 258 (38.3%) and 0.6, respectively (*p* values < 0.001 for both analysis), after the review process by the clinical committee and according to patients’ clinical evolution. A similar trend was observed for START criteria. Initially, in 247 (36.7%) patients one or more START criteria were detected while, after hospitalization, this percentage was significantly reduced to 15.7% (106 patients, *p* value < 0.001). The degree of reduction of STOPP criteria was similar among all participating centers, while START criteria were only substantially reduced in three out of five hospitals. These data are reflected in Table 1, along with the distribution of patients recorded in each of the hospitals participating in the study.

**Table 1:** Number of patients with any STOPP and/or START criteria and average of STOPP and/or START criteria per patient at admission and at discharge

Hospital	Total patients	Number of patients with any STOPP criteria at admission, N (%)	Number of patients with any STOPP criteria at discharge, N (%)	Number of STOPP criteria per patient at admission, mean [range]	Number of STOPP criteria per patient at discharge, mean [range]	Number of patients with any START criteria at admission, N (%)	Number of patients with any START criteria at discharge, N (%)	Number of START criteria per patient at admission, mean [range]	Number of START criteria per patient at discharge, mean [range]
A	87	56 (64.4)	9 (10.3)	1.2 [0; 5]	0.1 [0; 2]	11 (12.6)	11 (12.6)	0.1 [0; 1]	0.3 [0; 4]
B	162	122 (75.3)	44 (27.2)	1.3 [0; 5]	0.4 [0; 4]	51 (31.5)	17 (10.5)	0.5 [0; 3]	0.1 [0; 3]
C	216	184 (85.2)	106 (49.1)	2.3 [0; 8]	0.7 [0; 4]	104 (48.2)	18 (8.3)	0.6 [0; 4]	0.1 [0; 2]
D	101	53 (52.5)	39 (38.6)	1.0 [0; 6]	0.7 [0; 5]	40 (39.6)	23 (22.8)	0.6 [0; 3]	0.3 [0; 3]
E	108	78 (72.2)	60 (55.6)	1.7 [0; 6]	1.0 [0; 5]	41 (38.0)	37 (34.3)	0.5 [0; 4]	0.5 [0; 4]
Total	674	493 (73.2)	258 (38.3)	1.6 [0; 8]	0.6 [0; 5]	247 (36.7)	106 (15.7)	0.5 [0; 4]	0.2 [0; 4]

As shown in Supplementary Figure S1A, only 26.9% of patients were admitted without any STOPP criteria detected by the clinical committee. Oppositely, 43.6% of patients presented two or more PIM at the initial revision. After hospitalization, 61.7% of patients were discharged without STOPP criteria, a significant decrease compared to admission time point, particularly in those cases with two or more STOPP criteria, which only comprised the 14.3% of patients. A similar trend was observed for START criteria (Supplementary Figure S1B), although a smaller improvement was achieved, with an increase from 63.3% to 84.3% in the proportion of patients without any START criteria.

### Description of PIM assessed by the clinical committee during patients’ hospitalization

In total, 1077 PIM were registered by the clinical committee at patients’ admission, whereas only 394 PIM were detected at discharge, indicating a global reduction of 63.4%. For a more detailed description of PIM detected at admission and at discharge, a complete distribution of all STOPP criteria identified can be found at Supplementary Table S4. As a summary, the top 20 STOPP criteria with the highest percentage of detection at admission are reported in Table 2. Remarkably, three of the criteria accounted for a total of 44.1% of the items registered: D5 (benzodiazepines (BZD) administration for more than 4 weeks), 20.7%; A1 (prescription of any drug without evidence-based clinical indication, restricted to acid reducers), 12.8%; and K1 (prescription of BZD that increases risk of falls), 10.6%.

**Table 2:** Top 20 STOPP criteria at admission and discharge, ordered by descending % at admission

Criteria	Admission		Discharge		% of change^*^
	N	Col. %	N	Col. %	
D5: Benzodiazepines for ≥ 4 weeks	223	20.7	82	20.8	-63.2
A1: Acid reducer	138	12.8	114	28.9	-17.4
K1: Benzodiazepines	114	10.6	28	7.1	-75.4
A1: Any drug prescribed without an evidence-based clinical indication	63	5.9	15	3.8	-76.2
G5: Benzodiazepines with acute or chronic respiratory failure i.e. pO2 < 8.0 kPa ± pCO2 > 6.5 kPa	46	4.3	9	2.3	-80.4
B11: ACE inhibitors or Angiotensin Receptor Blockers in patients with hyperkalaemia	41	3.8	1	0.3	-97.6
A2: Any drug prescribed beyond the recommended duration, where treatment duration is well defined	36	3.3	6	1.5	-83.3
L2: Use of regular (as distinct from PRN) opioids without concomitant laxative	33	3.1	13	3.3	-60.6
A3: Any duplicate drug class prescription	30	2.8	12	3.1	-60.0
A1: Hypolipidemic drug	27	2.5	2	0.5	-92.6
K2: Neuroleptic drugs	24	2.2	12	3.1	-50.0
A1: Analgesic drug	22	2.0	14	3.6	-36.4
B8: Thiazide diuretic with current significant hypokalaemia, hyponatraemia, hypercalcaemia or with a history of gout	18	1.7	1	0.3	-94.4
B12: Aldosterone antagonists with concurrent potassium-conserving drugs without monitoring of serum potassium	17	1.6	2	0.5	-88.2
L1: Use of oral or transdermal strong opioids as first line therapy for mild pain	15	1.4	8	2.0	-46.7
A1: Aspirin	13	1.2	3	0.8	-76.9
B5: Amiodarone as first-line antiarrhythmic therapy in supraventricular tachyarrhythmia	12	1.1	4	1.0	-66.7
B6: Loop diuretic as first-line treatment for hypertension	12	1.1	4	1.0	-66.7
C1: Long-term aspirin at doses greater than 160 mg per day	12	1.1	2	0.5	-83.3
I1: Antimuscarinic drugs with dementia, or chronic cognitive impairment or narrow-angle glaucoma, or chronic prostatism	11	1.0	8	2.0	-27.3

^*^(N discharge – N admission) / N admission × 100

In terms of PIM detection at discharge, all criteria included in Table 2 were clearly reduced by the intervention of the clinical committee, ranging from 17.4% to 97.6% of decrease. Interestingly, criteria A1 applied to acid reducers, despite being the second mostly registered, resulted in the lowest percentage of reduction, with only 17.4%. On the opposite side, criteria B11 (ACE inhibitors or AR blockers in patients with hyperkalemia), B8 (thiazide diuretics with current significant hypokalemia, hyponatremia, hypercalcemia or gout history) and A1 restricted to hypolipidemic drugs reached more than 90% of reduction (Table 2).

**Fig. 1:**
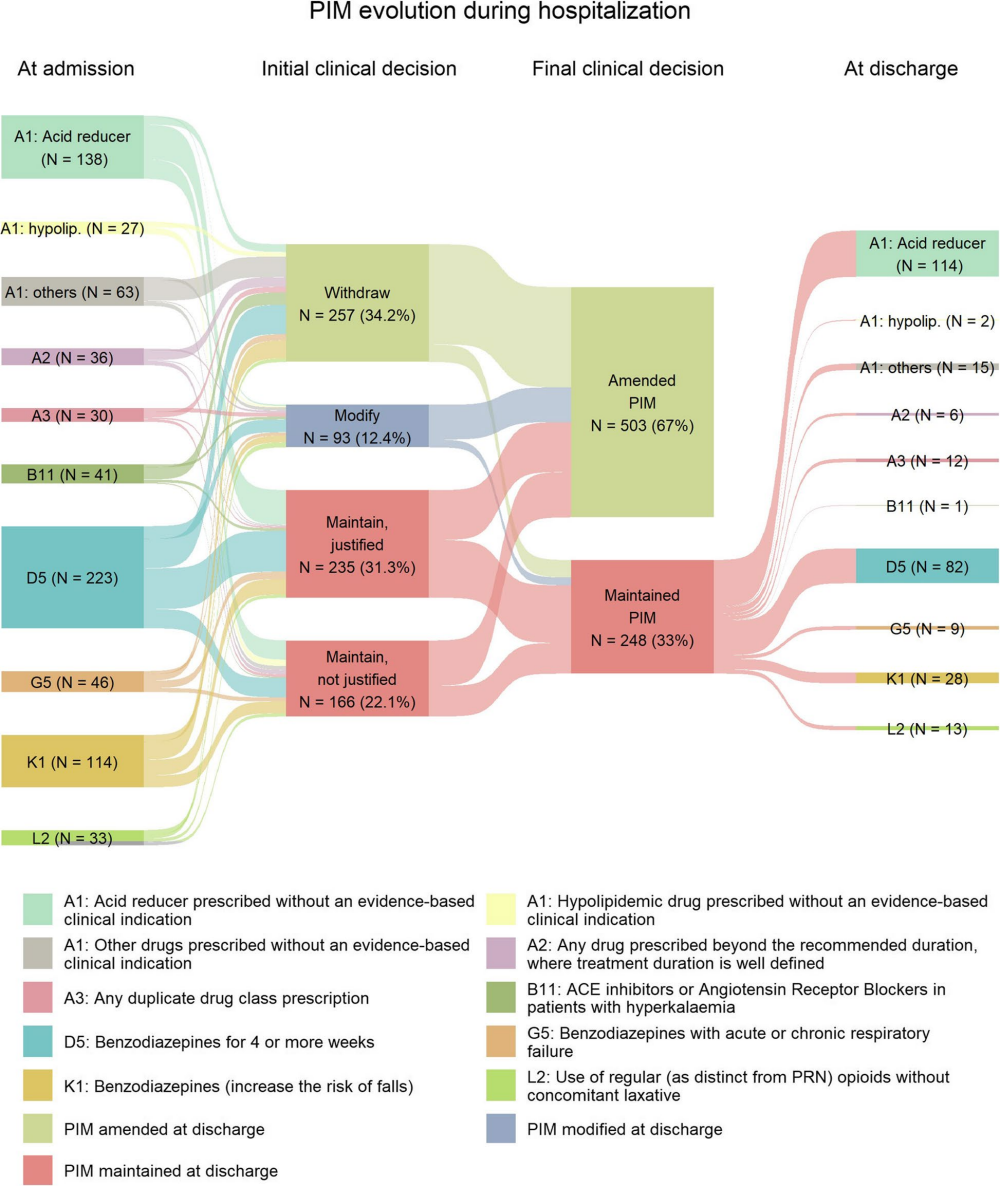
Decisions taken at the clinical committee review process on PIM evolve during patients’ hospitalization. Sankey diagram representing the distribution of clinical decisions on the top 10 most frequent PIM detected at admission. Number and percentage of prescriptions withdrawn, modified or maintained (with or without justification) derived from the initial clinical decisions are shown in the second column, while final clinical decisions are depicted in the third column as amended or maintained. Colors associated to each STOPP criteria are indicated in the legend

The top 10 STOPP criteria detected at admission were subjected to an evolution analysis during patients’ complete hospitalization. Out of a total of 751 prescriptions associated to these STOPP criteria, the clinical committee initially decided to withdraw 257 (34.2%) prescriptions and to modify 93 (12.4%) prescriptions. Contrarily, 401 (53.4%) prescriptions were maintained, 58.6% of them with clinical justification (Fig. 1). Later, the evaluation of the final clinical decisions revealed that 503 (67.0%) STOPP criteria were amended (i.e., the inappropriateness criteria were resolved either by drug withdrawal or posology adequacy). Instead, 248 (33.0%) STOPP criteria remained without modifications at patients’ discharge. Among these unmodified prescriptions, A1 restricted to acid reducers and D5 criteria prevailed as the most predominant, constituting the 46.0% and 33.1% of the prescriptions, respectively (Fig. 1).

### Description of PPO Assessed by the Clinical Committee during Patients’ Hospitalization

Regarding the analysis and further evaluation of PPO in our cohort, a total of 330 prescriptions with START criteria were reported at admission. According to the reduction of PIM mentioned before, PPO were also diminished in a large percentage at patients’ discharge. In this case, a 55.2% of reduction was achieved, registering only 148 PPO for the total of discharged patients. An extensive description of PPO detected at admission and at discharge, together with the full distribution of associated START criteria can be found in Supplementary Table S5. Additionally, the 20 criteria with a higher prevalence are summarized in Table 3. From this list, four criteria reached a percentage above 10%: E5 (vitamin D supplement in older people who are housebound or experiencing falls with osteopenia), 22.4%; H2 (laxative in patients receiving opioids regularly), 13.0%; A8 (appropriate beta-blocker with stable systolic heart failure), 11.2%; and A6 (ACE inhibitors with systolic heart failure and/or documented coronary artery disease), 10.6%.

**Table 3:** Top 20 START criteria at admission and discharge, ordered by descending % at admission

Criteria	Admission		Discharge		% of change^*^
	N	Col. %	N	Col. %	
E5: Vitamin D supplement in older people who are housebound or experiencing falls or with osteopenia (Bone Mineral Density T-score is > -1.0 but < -2.5 in multiple sites)	74	22.4	18	12.2	-75.7
H2: Laxatives in patients receiving opioids regularly	43	13.0	26	17.6	-39.5
A8: Appropriate beta-blocker (bisoprolol, nebivolol, metoprolol or carvedilol) with stable systolic heart failure	37	11.2	11	7.4	-70.3
A6: Angiotensin Converting Enzyme (ACE) inhibitor with systolic heart failure and/or documented coronary artery disease	35	10.6	15	10.1	-57.1
E3: Vitamin D and calcium supplement in patients with known osteoporosis and/or previous fragility fracture(s) and/or (Bone Mineral Density T-scores more than -2.5 in multiple sites)	20	6.1	15	10.1	-25.0
A1: Vitamin K antagonists or direct thrombin inhibitors or factor Xa inhibitors in the presence of chronic atrial fibrillation	18	5.5	5	3.4	-72.2
C2: Non-TCA antidepressant drug in the presence of persistent major depressive symptoms	14	4.2	1	0.7	-92.9
A7: Beta-blocker with ischaemic heart disease	13	3.9	4	2.7	-69.2
E2: Bisphosphonates and vitamin D and calcium in patients taking long-term systemic corticosteroid therapy	10	3.0	11	7.4	10.0
A3: Antiplatelet therapy (aspirin or clopidogrel or prasugrel or ticagrelor) with a documented history of coronary, cerebral or peripheral vascular disease	8	2.4	3	2.0	-62.5
A4: Antihypertensive therapy where systolic blood pressure consistently > 160 mmHg and/or diastolic blood pressure consistently > 90 mmHg; if systolic blood pressure > 140 mmHg and /or diastolic blood pressure > 90 mmHg, if diabetic	8	2.4	1	0.7	-87.5
C3: Acetylcholinesterase inhibitor (e.g. donepezil, rivastigmine, galantamine) for mild-moderate Alzheimer's dementia or Lewy Body dementia (rivastigmine)	8	2.4	6	4.1	-25.0
E4: Bone anti-resorptive or anabolic therapy (e.g. bisphosphonate, strontium ranelate, teriparatide, denosumab) in patients with documented osteoporosis, where no pharmacological or clinical status contraindication exists (Bone Mineral Density T-scores—> 2.5 in multiple sites) and/or previous history of fragility fracture(s)	7	2.1	4	2.7	-42.9
D1: Proton Pump Inhibitor with severe gastro-oesophageal reflux disease or peptic stricture requiring dilatation	5	1.5	2	1.4	-60.0
F1: ACE inhibitor or Angiotensin Receptor Blocker (if intolerant of ACE inhibitor) in diabetes with evidence of renal disease i.e. dipstick proteinuria or microalbuminuria (> 30 mg/24 h) with or without serum biochemical renal impairment	4	1.2	2	1.4	-50.0
G2: 5-alpha reductase inhibitor with symptomatic prostatism, where prostatectomy is not considered necessary	4	1.2	5	3.4	25.0
A5: Statin therapy with a documented history of coronary, cerebral or peripheral vascular disease, unless the patient's status is end-of-life or age is > 85 years	3	0.9	4	2.7	33.3
B2: Regular inhaled corticosteroid for moderate-severe asthma or COPD, where FEV1 < 50% of predicted value and repeated exacerbations requiring treatment with oral corticosteroids	3	0.9	2	1.4	-33.3
E6: Xanthine-oxidase inhibitors (e.g. allopurinol, febuxostat) with a history of recurrent episodes of gout	3	0.9	2	1.4	-33.3
A2: Aspirin (75 mg – 160 mg once daily) in the presence of chronic atrial fibrillation, where Vitamin K antagonists or direct thrombin inhibitors or factor Xa inhibitors are contraindicated	2	0.6	0	0.0	-100.0

^*^(N discharge – N admission) / N admission × 100

The analysis of PPO at discharge revealed that, differentially from PIM data, not all START criteria were reduced. However, we reduced the proportion of inappropriateness in 17 out of 20 items. We found that the START criteria with a higher percentage of improvement do not correspond to any of the most frequent ones. In this sense, we should emphasize that criteria C2 (non-TCA antidepressant drug in the presence of persistent major depressive symptoms) and A4 (antihypertensive therapy in high blood pressure) reached drastic reduction rates of 92.9% and 87.5%, respectively (Table 3).

**Fig. 2:**
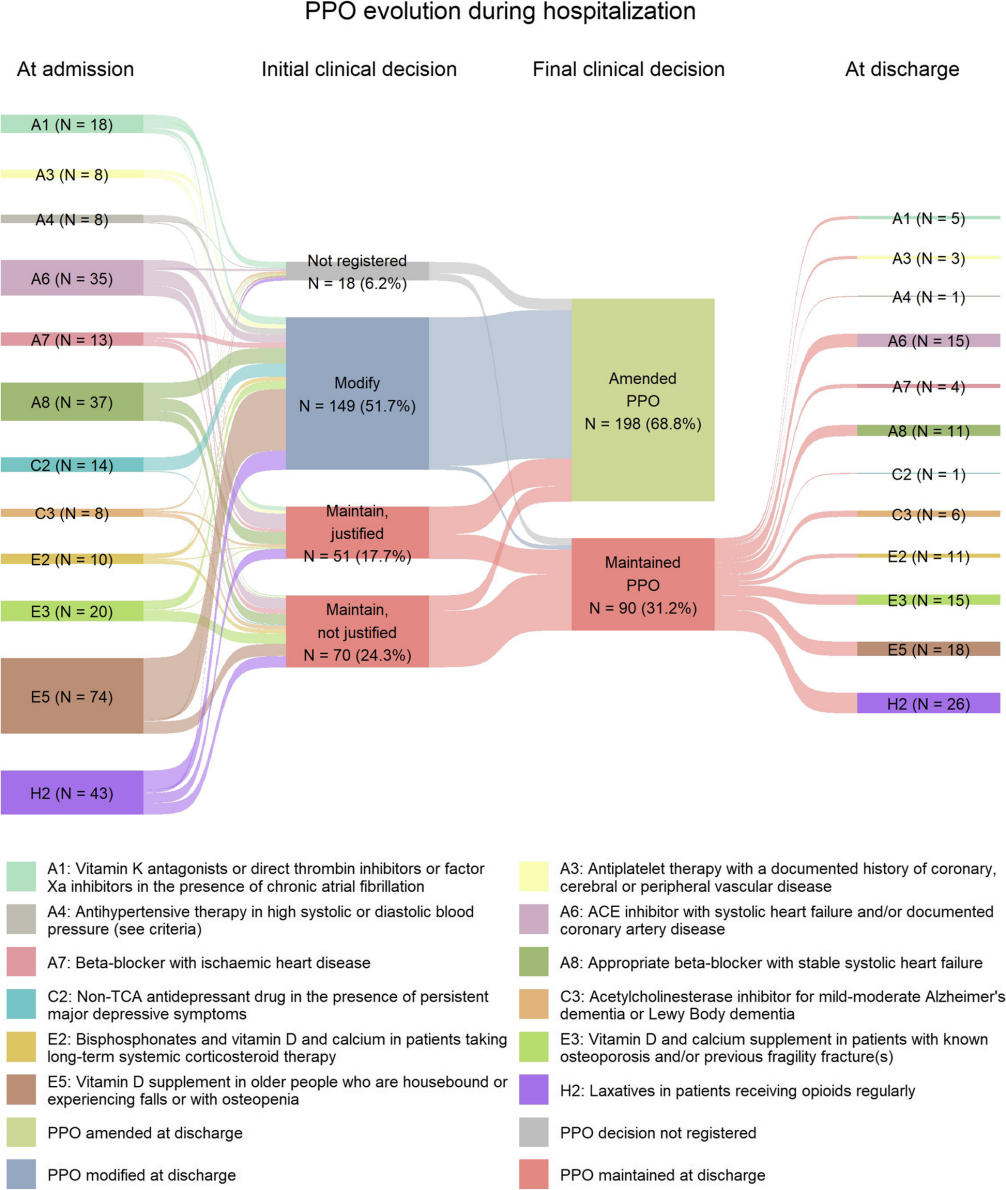
Decisions taken at the clinical committee review process on PPO evolve during patients’ hospitalization. Sankey diagram representing the distribution of clinical decisions on the top 10 most frequent PPO detected at admission. Number and percentage of prescriptions modified or maintained (with or without justification) derived from the initial clinical decisions are shown in the second column, while final clinical decisions are depicted in the third column as amended or maintained. In 18 START criteria the initial clinical decision was not registered. Colors associated to each START criteria are indicated in the legend

Again, we performed an evolution analysis during hospitalization of the top 10 START criteria detected at admission. In this case, the clinical committee agreed to initiate 149 (51.7%) prescriptions associated to any START criteria, while 121 (42.0%) PPO registered at admission were not modified at the initial decision. Most of these decisions (70 out of 121, 57.9%) were considered as not justified during the initial days of admission (Fig. 2). When assessing final clinical decisions, the proportion of START criteria amended slightly increased up to 198 (68.8%) prescriptions, whereas in 90 (31.2%) PPO the initial decision prevailed. Interestingly, the criteria accounting for the higher number of unmodified decisions were H2 (26, 28.9%) and E5 (18, 20.0%) (Fig. 2).

## Discussion

### Main results

The large cohort of patients included in this study presents a high incidence of PIDP according to STOPP/START criteria, as it could be expected from the characteristics of the population analysed, mainly because of their comorbidities and polypharmacy. Data collected from the clinical committee review process in the five hospitals participating in the MoPIM study, demonstrate that PIDP detection, review and follow-up for each patient by a multidisciplinary team has a strong benefit in terms of reducing prescription inadequacy in our cohort.

### Comparison of our results with other studies

The optimization of medical prescription in older patients, with the final goal of diminishing PIDP incidence, is one of the main concerns among clinical teams. In fact, several studies retrospectively comparing the incidence of PIDP between patients’ admission and discharge have been reported in the last years [26–30]. Nevertheless, they were performed in a retrospective manner and provide contradictory results. Some publications indicate that the lack of clinical intervention during hospitalization causes an increase in the total number of PIDP. For instance, Perpetuo et al. reported that, at admission, 49.4% of patients presented at least one STOPP criteria and this value augmented up to 62.2% at discharge [26]. Similar results were published by Abukhalil et al., reporting an increase of 8.1% between admission and discharge, in a younger patients’ cohort with lower degree of polypharmacy and comorbidity Charlson Index compared to our cohort [27]. Instead, other studies show a surprising drastic reduction of PIDP, even without mentioning a clinical review process during patients’ stay [29, 30]. Therefore, we would like to stress the importance of performing prospective studies with well-defined criteria and methodology to analyze the evolution of PIDP during patients’ hospitalization.

On the other hand, there are few studies describing how the intervention of multidisciplinary teams (including physicians, pharmacists and nurses) improves prescription adequacy during hospitalization in older patients’ cohorts. Importantly, the MoPIM prospective study includes a large cohort of patients, with more than 670 individuals comprising a two-year period from five different centers within the same country. In this sense, few prospective reports aiming to analyze geriatric team interventions have been published [31–33]. In these studies, the average age of patients included is above 80 years old, with most females. Therefore, although STOPP/START criteria are conceptually developed to be applied in ≥ 65-year-old patients, the actual cohorts to analyze are much older, reflecting the high percentage of octogenarians in Western society.

Unfortunately, these prospective studies do not share the same methodology with MoPIM study and account for smaller cohorts of around 100–200 patients. Specifically, Hanou et al*.* published data on a cohort with a wide majority of psychogeriatric patients, whereas version 1 of STOPP/START criteria was employed [12, 31]. Moreover, Farhat et al*.* divided their cohort in two subgroups, in order to compare STOPP/START with PIM-Check criteria [32, 34]. Finally, study by Magallón-Martínez et al*.* analyzed pharmaceutical interventions based not only in STOPP/START criteria, but also in Beers, Priscus and LESS-CHRON criteria [10, 33, 35]. In consequence, no solid conclusions can be extracted from the extrapolation of our data with these previous reports.

In MoPIM study, each clinical review committee counts with the intervention of a clinical pharmacist, who identifies all PIDP associated to patients’ medication and promotes the reduction of inappropriate prescriptions. Remarkably, previous reports have shown that including pharmacists in multidisciplinary geriatric teams provides more benefits than isolated pharmacists’ interventions [20]. Moreover, a recent report has demonstrated that the intervention of a clinical pharmacist can improve medical prescriptions by diminishing the total PIDP incidence in a cohort of older patients admitted for acute hip fracture [36]. In a small intervention cohort of 59 patients, Leguillon et al. obtained a reduction around 65% of total PIDP between admission and discharge. This effect clearly improves the comparison of the control group that, without the involvement of a clinical pharmacist, achieves a very slight prescription adequacy in terms of PIDP incidence [36].

In our cohort, an average of 2.1 STOPP/START criteria per patient is detected at admission. These data are similar to previous studies, despite being performed in a cohort of psychogeriatric patients or in a medium-stay unit [15, 31, 37]. Oppositely, our results show a lower identification of PIDP/patient when compared to a cohort of individuals admitted to a geriatric perioperative care unit [36]. Remarkably, data from Farhat et al. reveal a lower detection of PIDP based on STOPP/START criteria, with only 0.8 PIDP/patient at admission [32]. However, the use of PIM-Check criteria in this same study results in an average of 2.3 PIDP/patient, indicating that the identification of PIDP can vary depending on the criteria used. An example of this inconsistency among different criteria is found in the publication from Grina et al., where the use of EU-PIM and Beers criteria identified less PIDP compared to STOPP/START [37].

In our cohort, the PIM analysis demonstrated a high incidence of D5 and K1, both of them STOPP criteria related to an inappropriate use of BZD. In fact, these items ranked first and third among the top 20 most frequently identified. In this direction, several studies have reported that the inadequate prescription of BZD, according to STOPP criteria, are found among the most frequent PIM [31, 32, 38]. However, other studies did not find use of BZD as any of the most detected PIM [26], probably due to the struggles in determining prescription adequacy retrospectively. Interestingly, through our clinical committee review process, we have been able to reduce the prescription of BZD between 63 and 75%, depending on the criteria, in the internal medicine and the geriatric services. In contrast, other studies exclusively involving patients admitted in a psychogeriatrics unit resulted in no reduction of BZD use, indicating the difficulties to deprescribe these drugs in this specific hospital unit [31].

The inappropriate use of PPI was encountered as the second most common STOPP criteria in this study, in parallel to previous data that reported a high incidence of incorrect PPI prescription [39, 40]. As it is widely known, an elevated use of PPI can result in adverse effects such as bone fractures, hypomagnesemia, *C. difficile* infections, dementia, respiratory infections and community-acquired pneumonia [25, 41]. With the aim of preventing all these side effects, it is essential to carefully review PPI prescriptions and deprescribe them in case of inappropriate indication [42].

### Clinical interpretation of results

We observed that STOPP criteria with the highest levels of adequacy in MoPIM study were those associated to the onset of adverse effects, mostly affecting the cardiovascular system. For instance, hyperkalemia linked to inadequate prescription of ACE inhibitors or AR blockers (B11 criteria) or the use of thiazide diuretics with current hypokalemia, hyponatremia, hypercalcaemia, or gout history (B8 criteria) reached 97.6% and 94.4% of deprescription, respectively, when the withdrawal of these drugs was suggested. Additionally, unnecessary prescribed lipid-lowering drugs were withdrawn in more than 90% of cases (STOPP criteria A1), given that the benefit-risk balance of statin use as prevention of cardiovascular disease is controversial in older patients [43]. Contrarily, most of prescriptions initiated upon PPO detection comprise the use of drugs with immediate benefits in the treatment of common pathologies in older patients, such as major depression, hypertension, osteopenia, chronic atrial fibrillation and heart failure. Therefore, according to our data, physicians are more prone to deprescribe drugs when patients’ safety is already challenged by a specific adverse effect, as well as to initiate drug prescriptions that improve patients’ quality of life and prognosis.

### Impact and barriers of the clinical committee review process

The MoPIM study was not only designed to detect PIDP at patient’s admission and discharge, but also to evaluate how the degree of inappropriately prescribed drugs evolved during patients’ hospitalization. In this sense, the clinical decisions from the reviewing committee resulted in a deprescription or posology adequacy in 67.0% of PIM at patients’ discharge, although only 46.6% recommendations had been accepted after the initial decision. Regarding PPO, the initial decision suggested to start omitted prescriptions in 51.7% of cases, while this ratio increased up to 68.8% of acceptance at discharge. Therefore, these data indicate that chronic patients evolve during hospital stay and, in most cases, the adequacy of prescriptions may be easier at discharge, rather than at admission, when they have just experienced an acute exacerbation of their conditions. Apart from patients’ own changes in clinical evolution, it has been demonstrated that the inclusion of a pharmacist in a clinical team improves the accomplishment of prescription adequacy criteria [44].

However, reaching a full acceptance of the agreements taken by the clinical review committee in terms of deprescription is still utopic, due to several barriers interfering in this process. As revised by Peat et al*.*, those barriers are related to the organization of healthcare settings (consultation constraints or perceived hierarchies), to communication transparency (sharing decisions and information), as well as to patients’ habits and concerns (fears of negative consequences of deprescribing) [45]. In the particular case of BZD, communication between patients and physicians is essential to prevent reluctance to its deprescription [46], which is difficult to achieve during hospital admission.

The clinical committee review, with the guidance of STOPP/START explicit criteria, has promoted the identification and decrease of PIDP, which have been also linked to an increased risk of hospital readmission [47]. Specifically, in RESORT study, PIDP related to the inappropriate use of central nervous system drugs or to an elevated fall risk were found to be significantly associated with 30-day hospital readmission [47]. In our study, we have been able to largely reduce the inadequate prescriptions related to D5 and K1 criteria, referring to the use of BZD and its correlation with fall risk in older people. Additionally, the improvement in detection and amendment of PPO can be related to an enhancement in patient’s independence in daily instrumental activities, despite lack of association with clinical outcomes [47]. In fact, a recent Cochrane systematic review of 25 trials and more than 15,000 patients, with a vast majority of older adults with polypharmacy, determined that medication review reduces hospital readmission and may prevent emergency room contacts [48].

### PIDP and polypharmacy

In the last decade, polypharmacy has emerged as one of the major concerns among clinical teams in care of older adults [49]. In our cohort, patients present an average of 10.6 prescribed drugs at admission, higher than other comparable studies [31, 32]. However, despite patients are admitted due to an exacerbation or decompensation of their chronic pathologies, the total number of prescriptions is maintained at discharge (with an average of 11.0 drugs/patient). In consequence, polypharmacy is not decreased, whereas prescription adequacy is improved through the reduction of PIDP, emphasizing the importance of the clinical committee review process.

In fact, the increase of polypharmacy in older patients has demonstrated to be a risk factor for more hospitalizations and emergency room visits [50]. In consequence, it is necessary to consider deprescription in order to prevent inappropriate polypharmacy. However, these actions will probably require a shift in prescribing culture [49]. Our data indicate that this process may be successfully led by a clinical pharmacist, as demonstrated in a recent meta-analysis, reporting that pharmaceutical interventions significantly reduce the incidence of PIM in older patients, along with polypharmacy and 30-day readmission rate [51].

### Strengths and limitations of the study

The multicenter approach of the protocol design is, undoubtedly, one of the most valuable strengths, particularly for the use of the second version of STOPP/START criteria, released just a few months before initiating the study [13]. Remarkably, it embraces different regions across Spain, only including hospitals where clinical pharmacists have a complete integration in multidisciplinary teams, together with geriatricians and internal medicine physicians [24]. As thoroughly described across this section, we would like to highlight the prospective design of the protocol, which has allowed a more accurate and close monitoring of the decisions taken on pharmacotherapy adequacy during patients’ hospitalization. Moreover, during the clinical committee review process, newly prescribed medication of patients due to the acute condition has been considered, along with their chronic therapy, thus reflecting previous patients’ management at primary care settings. Finally, we analyze how the acceptance of PIDP adequacy recommendations varies between admission of patients and discharge, reflecting the importance of a complete supervision of PIDP during the whole hospital stay.

Nevertheless, the design of this study displays some limitations, such as a potential variability in STOPP/START criteria application among different centers. This bias may be caused by the personal point of view of each professional involved or the available tools for medication review. A further bias in the study can exist due to the exclusion of patients who died during hospital stay. Importantly, none of the patients’ death was directly linked to a particular drug inadequacy. Given that this study was conceived as a non-comparative study, the data presented have not been compared to a control group. Moreover, the specific clinical reasons for maintaining some PIM or PPO detected at discharge were not registered. In addition, the protocol does not include further patients’ follow-up after discharge, which would be really useful to analyze whether PIDP adequacy is maintained during subsequent months, and how our interventions are translated into clinical relevance, such as affecting patients’ morbidity and mortality.

Of note, an update of the STOPP/START criteria (version 3) has been recently published [52], but it was not available at the time this study was designed and executed. In this new version, the number of items has been enlarged up to 190 criteria, 67% more than in previous version. The higher number of criteria will probably improve the detection of PIDP in a more accurate manner since they consider the availability of several new medications for acute and chronic treatments, although it will difficult the review process by clinical teams, converting the assistance of informatics tools in indispensable.

## Conclusions

The study corroborates an elevated incidence of polypharmacy and PIDP in our patients’ cohort, especially PIM associated to the inappropriate use of BZD and acid reducers. Importantly, the clinical committee review process succeeded in identifying and reducing the degree of prescription inadequacy, for both STOPP and START criteria. Moreover, we have observed that the acceptance of clinical pharmacist recommendations increases at patients’ discharge, compared to the initial clinical decisions upon patients’ admission. Globally, pharmacotherapy review through the application of explicit STOPP/START criteria improve prescription adequacy in older patients with high degree of multimorbidity and polypharmacy.

### Supplementary Information

Supplementary Material 1.

Supplementary Material 2.

## Data Availability

The data presented in this study are openly available in Zenodo at 10.5281/zenodo.7371151.

## Abbreviations

ADR: Adverse drug reaction
MoPIM: Morbidity, potentially inappropriate medication
PIDP: Potentially inappropriate drug prescription
PIM: Potentially inappropriate medication
PPO: Potential prescribing omission
