# Supplementary figures and images for: Multidisciplinary medication review during older patient hospitalization according to STOPP/START criteria reduces potentially inappropriate prescriptions: MoPIM cohort study
Source: BMC Geriatr. 2024 Jul 8;24:584. doi: 10.1186/s12877-024-05185-w (PMC11232270; doi:10.1186/s12877-024-05185-w)

**A** Patients with any STOPP

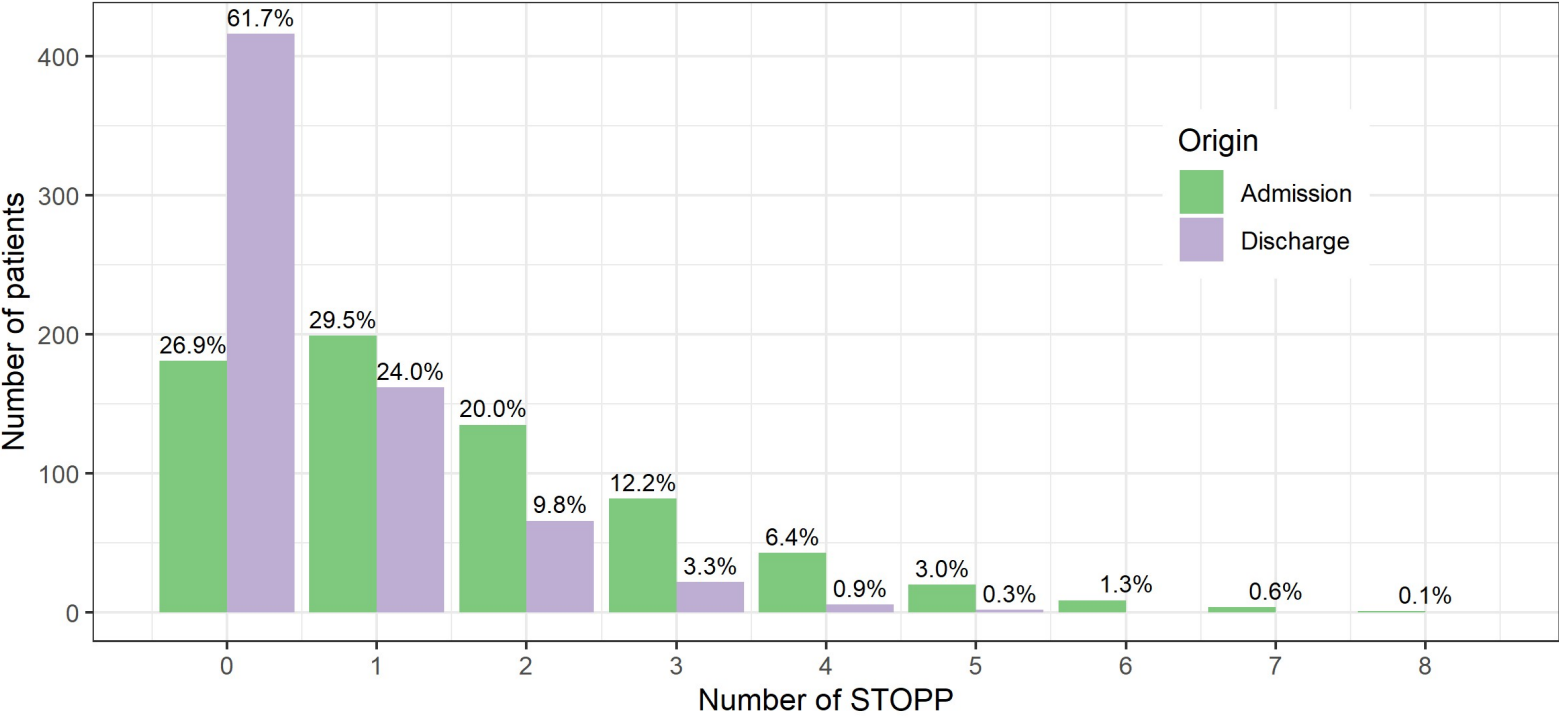

**B** Patients with any START

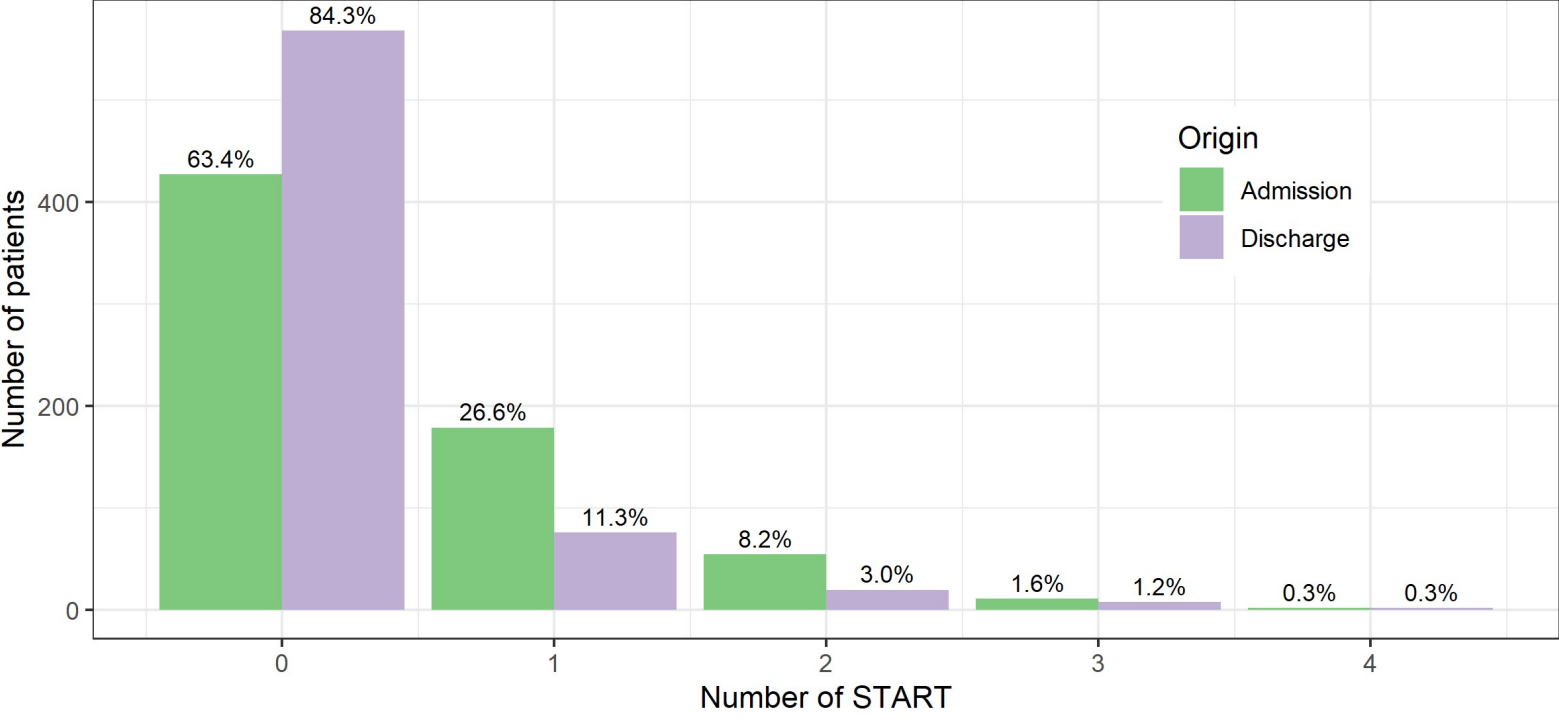

Supplement: Supplementary file 1 — Supplementary Material 1. [file 12877_2024_5185_MOESM1_ESM.pdf]
