# Supplementary material for: Multidisciplinary medication review during older patient hospitalization according to STOPP/START criteria reduces potentially inappropriate prescriptions: MoPIM cohort study
Source: BMC Geriatr. 2024 Jul 8;24:584. doi: 10.1186/s12877-024-05185-w (PMC11232270; doi:10.1186/s12877-024-05185-w)
Supplement: Supplementary file 2 — Supplementary Material 2. [file 12877_2024_5185_MOESM2_ESM.docx]

**Supplementary Table S1. Sociodemographic and clinical variables of the patients included in the study.**

| Sociodemographic and clinical variables | | N | % | 95% CI |
| --- | --- | --- | --- | --- |
| Age | < 70 | 32 | 4.8 | 3.4 - 6.6 |
|  | 70-74 | 42 | 6.2 | 4.6 - 8.3 |
|  | 75-79 | 70 | 10.4 | 8.3 - 13.0 |
|  | 80-84 | 164 | 24.3 | 21.2 - 27.7 |
|  | 85-89 | 220 | 32.6 | 29.2 - 36.3 |
|  | 90-94 | 121 | 18.0 | 15.2 - 21.0 |
|  | >= 95 | 25 | 3.7 | 2.5 - 5.4 |
| Sex | Female | 356 | 52.8 | 49.0 - 56.6 |
|  | Male | 318 | 47.2 | 43.4 - 51.0 |
| Barthel index | < 20 | 70 | 10.4 | 8.3 - 12.9 |
|  | 20-35 | 65 | 9.6 | 7.6 - 12.1 |
|  | 40-55 | 112 | 16.6 | 14.0 - 19.6 |
|  | 60-95 | 277 | 41.1 | 37.4 - 44.9 |
|  | 100 | 150 | 22.3 | 19.3 - 25.6 |
| Updated Charlson Comorbidity Index, age-adjusted | 2-5 | 136 | 20.2 | 17.3 - 23.4 |
|  | 6-8 | 376 | 55.8 | 52.0 - 59.5 |
|  | 9-14 | 162 | 24.0 | 21.0 - 27.4 |
| Household | Alone | 114 | 16.9 | 14.3 - 19.9 |
|  | Nursing home | 85 | 12.6 | 10.3 - 15.3 |
|  | With relatives/other people | 475 | 70.5 | 66.9 - 73.8 |
| Length of stay | 1-5 | 80 | 11.9 | 9.6 - 14.5 |
|  | 6-10 | 240 | 35.6 | 32.1 - 39.3 |
|  | 11-15 | 149 | 22.1 | 19.1 - 25.4 |
|  | 16-20 | 88 | 13.0 | 10.7 - 15.8 |
|  | 21-30 | 64 | 9.5 | 7.5 - 11.9 |
|  | > 30 | 53 | 7.9 | 6.1 - 10.1 |
| Destination at discharge | Another hospital | 101 | 15.0 | 12.5 - 17.9 |
|  | Home | 468 | 69.4 | 65.9 - 72.8 |
|  | Nursing home | 105 | 15.6 | 13.0 - 18.5 |

**Supplementary Table S2. Chronic active conditions of the patients included in the study, ordered by descending prevalence.**

| Chronic conditions | Total (674) | | |
| --- | --- | --- | --- |
|  | N | % | 95% CI |
| Hypertension | 548 | 81.31 | 78.19 - 84.07 |
| Heart failure | 401 | 59.50 | 55.75 - 63.14 |
| Cardiac arrhythmia | 380 | 56.38 | 52.61 - 60.08 |
| Degenerative arthropathy | 340 | 50.45 | 46.68 - 54.21 |
| Dyslipidaemia | 328 | 48.66 | 44.91 - 52.43 |
| Anaemia | 300 | 44.51 | 40.80 - 48.28 |
| Chronic renal insufficiency | 288 | 42.73 | 39.05 - 46.50 |
| Chronic obstructive pulmonary disease | 248 | 36.80 | 33.24 - 40.50 |
| Non-ischaemic heart disease | 218 | 32.34 | 28.92 - 35.97 |
| Diabetes mellitus without organ damage | 188 | 27.89 | 24.64 - 31.40 |
| Cerebrovascular disease | 172 | 25.52 | 22.37 - 28.94 |
| Obesity | 170 | 25.22 | 22.09 - 28.64 |
| Dementia | 158 | 23.44 | 20.40 - 26.79 |
| Varicose veins | 147 | 21.81 | 18.86 - 25.08 |
| Fracture (excluding hip) | 128 | 18.99 | 16.21 - 22.12 |
| Gout | 121 | 17.95 | 15.24 - 21.03 |
| Thyroid disease | 120 | 17.80 | 15.10 - 20.87 |
| Diabetes mellitus with organ damage | 117 | 17.36 | 14.69 - 20.40 |
| Coronary ischaemic disease | 107 | 15.88 | 13.31 - 18.83 |
| Myocardial infarction | 101 | 14.99 | 12.49 - 17.88 |
| Neoplasia | 101 | 14.99 | 12.49 - 17.88 |
| Osteoporosis | 95 | 14.09 | 11.67 - 16.93 |
| Peripheral arteriopathy | 89 | 13.20 | 10.86 - 15.97 |
| Gastroesophageal reflux disease | 87 | 12.91 | 10.59 - 15.65 |
| Asthma | 78 | 11.57 | 9.37 - 14.21 |
| Gallstones | 74 | 10.98 | 8.84 - 13.56 |
| Vertigo | 72 | 10.68 | 8.57 - 13.24 |
| Hip fracture | 63 | 9.35 | 7.37 - 11.78 |
| Drug related conditions | 59 | 8.75 | 6.85 - 11.13 |
| Sleep apnoea | 58 | 8.61 | 6.72 - 10.96 |
| Peripheral neuropathy | 53 | 7.86 | 6.06 - 10.14 |
| Inflammatory osteoarticular disease | 46 | 6.82 | 5.16 - 8.98 |
| Ulcerative disease | 42 | 6.23 | 4.64 - 8.32 |
| Mild liver disease | 32 | 4.75 | 3.38 - 6.63 |
| Parkinson's disease | 32 | 4.75 | 3.38 - 6.63 |
| Haematological diseases | 32 | 4.75 | 3.38 - 6.63 |
| Autoimmune and/or connective tissue disease | 29 | 4.30 | 3.01 - 6.11 |
| Central nervous system neurological disease | 27 | 4.01 | 2.77 - 5.77 |
| Severe liver disease | 19 | 2.82 | 1.81 - 4.36 |
| Amputation | 14 | 2.08 | 1.24 - 3.46 |
| Urinary tract stones | 11 | 1.63 | 0.91 - 2.90 |
| Non-schizophrenic mental disorders | 11 | 1.63 | 0.91 - 2.90 |
| Irritable bowel syndrome | 10 | 1.48 | 0.81 - 2.71 |
| Tuberculosis | 9 | 1.34 | 0.70 - 2.52 |
| Other neurological pathologies | 9 | 1.34 | 0.70 - 2.52 |
| Pancreas disease | 8 | 1.19 | 0.60 - 2.32 |
| Fibromyalgia | 8 | 1.19 | 0.60 - 2.32 |
| Migraine | 4 | 0.59 | 0.23 - 1.52 |
| Schizophrenia | 3 | 0.45 | 0.15 - 1.30 |
| Post-traumatic stress disorder | 2 | 0.30 | 0.08 - 1.08 |

**Supplementary Table S3. Geriatric syndromes conditions of the patients included in the study, ordered by descending prevalence.**

| Geriatric syndromes | Total (674) | | |
| --- | --- | --- | --- |
|  | N | % | 95% CI |
| Polypharmacy | 535 | 79.38 | 76.16 - 82.26 |
| Frailty | 406 | 60.24 | 56.49 - 63.86 |
| Incontinence (urinary/faecal) | 380 | 56.38 | 52.61 - 60.08 |
| Chronic pain | 356 | 52.82 | 49.04 - 56.56 |
| Constipation | 311 | 46.14 | 42.41 - 49.92 |
| Sleep disorders/Insomnia | 296 | 43.92 | 40.22 - 47.69 |
| Sensory deficit | 283 | 41.99 | 38.32 - 45.75 |
| Instability/falls | 262 | 38.87 | 35.27 - 42.61 |
| Depression or anxiety | 237 | 35.16 | 31.65 - 38.84 |
| Acute confusional syndrome/delirium | 201 | 29.82 | 26.49 - 33.38 |
| Cognitive/Intellectual impairment | 198 | 29.38 | 26.06 - 32.92 |
| Immobility | 155 | 23.00 | 19.98 - 26.32 |
| Dysphagia | 136 | 20.18 | 17.32 - 23.37 |
| Malnutrition | 132 | 19.58 | 16.76 - 22.75 |
| Pressure ulcers | 74 | 10.98 | 8.84 - 13.56 |

**Supplementary Table S4. All STOPP criteria identified at admission and discharge.**

| Criteria | Admission | | Discharge | | % of change |
| --- | --- | --- | --- | --- | --- |
|  | N | % | N | % |  |
| A1: Any drug prescribed without an evidence-based clinical indication (other) | 63 | 5.9 | 15 | 3.8 | -76.2 |
| A1: Aspirin | 13 | 1.2 | 3 | 0.8 | -76.9 |
| A1: Acid reducer | 138 | 12.8 | 114 | 28.9 | -17.4 |
| A1: Analgesic drug | 22 | 2.0 | 14 | 3.6 | -36.4 |
| A1: Antihypertensive drug | 9 | 0.8 | 0 | 0 | -100 |
| A1: Hypolipidemic drug | 27 | 2.5 | 2 | 0.5 | -92.6 |
| A2: Any drug prescribed beyond the recommended duration, where treatment duration is well defined | 36 | 3.3 | 6 | 1.5 | -83.3 |
| A3: Any duplicate drug class prescription | 30 | 2.8 | 12 | 3.1 | -60.0 |
| B1: Digoxin for heart failure with normal systolic ventricular function | 5 | 0.5 | 1 | 0.3 | -80.0 |
| B11: ACE inhibitors or Angiotensin Receptor Blockers in patients with hyperkalaemia | 41 | 3.8 | 1 | 0.3 | -97.6 |
| B12: Aldosterone antagonists with concurrent potassium-conserving drugs without monitoring of serum potassium | 17 | 1.6 | 2 | 0.5 | -88.2 |
| B2: Verapamil or diltiazem with NYHA Class III or IV heart failure | 5 | 0.5 | 1 | 0.3 | -80.0 |
| B3: Beta-blocker in combination with verapamil or diltiazem | 1 | 0.1 | 0 | 0 | -100 |
| B4: Beta blocker with bradycardia, type II heart block or complete heart block | 3 | 0.3 | 0 | 0 | -100 |
| B5: Amiodarone as first-line antiarrhythmic therapy in supraventricular tachyarrhythmia | 12 | 1.1 | 4 | 1.0 | -66.7 |
| B6: Loop diuretic as first-line treatment for hypertension | 12 | 1.1 | 4 | 1.0 | -66.7 |
| B7: Loop diuretic for dependent ankle oedema without clinical, biochemical evidence or radiological evidence of heart failure, liver failure, nephrotic syndrome or renal failure | 7 | 0.7 | 3 | 0.8 | -57.1 |
| B8: Thiazide diuretic with current significant hypokalaemia, hyponatraemia, hypercalcaemia or with a history of gout | 18 | 1.7 | 1 | 0.3 | -94.4 |
| B9: Loop diuretic for treatment of hypertension with concurrent urinary incontinence | 4 | 0.4 | 2 | 0.5 | -50.0 |
| C1: Long-term aspirin at doses greater than 160mg per day | 12 | 1.1 | 2 | 0.5 | -83.3 |
| C10: NSAID and vitamin K antagonist, direct thrombin inhibitor or factor Xa inhibitors in combination | 5 | 0.5 | 2 | 0.5 | -60.0 |
| C2: Aspirin with a past history of peptic ulcer disease without concomitant PPI | 4 | 0.4 | 1 | 0.3 | -75.0 |
| C3: Aspirin, clopidogrel, dipyridamole, vitamin K antagonists, direct thrombin inhibitors or factor Xa inhibitors with concurrent significant bleeding risk | 4 | 0.4 | 1 | 0.3 | -75.0 |
| C4: Aspirin plus clopidogrel as secondary stroke prevention, unless the patient has a coronary stent(s) inserted in the previous 12 months or concurrent acute coronary syndrome or has a high grade symptomatic carotid arterial stenosis | 3 | 0.3 | 1 | 0.3 | -66.7 |
| C5: Aspirin in combination with vitamin K antagonist, direct thrombin inhibitor or factor Xa inhibitors in patients with chronic atrial fibrillation | 8 | 0.7 | 3 | 0.8 | -62.5 |
| C6: Antiplatelet agents with vitamin K antagonist, direct thrombin inhibitor or factor Xa inhibitors in patients with stable coronary, cerebrovascular or peripheral arterial disease | 2 | 0.2 | 2 | 0.5 | 0.0 |
| C8: Vitamin K antagonist, direct thrombin inhibitor or factor Xa inhibitors for first deep venous thrombosis without continuing provoking risk factors for > 6 months | 2 | 0.2 | 0 | 0 | -100 |
| C9: Vitamin K antagonist, direct thrombin inhibitor or factor Xa inhibitors for first pulmonary embolus without continuing provoking risk factors for > 12 months | 3 | 0.3 | 0 | 0 | -100 |
| D1: TriCyclic Antidepressants (TCAs) with dementia, narrow angle glaucoma, cardiac conduction abnormalities, prostatism, or prior history of urinary retention | 7 | 0.7 | 2 | 0.5 | -71.4 |
| D10: Neuroleptics as hypnotics, unless sleep disorder is due to psychosis or dementia | 2 | 0.2 | 2 | 0.5 | 0.0 |
| D11: Acetylcholinesterase inhibitors with a known history of persistent bradycardia, heart block or recurrent unexplained syncope or concurrent treatment with drugs that reduce heart rate | 1 | 0.1 | 0 | 0 | -100 |
| D14: First-generation antihistamines | 3 | 0.3 | 1 | 0.3 | -66.7 |
| D2: Initiation of TriCyclic Antidepressants (TCAs) as first-line antidepressant treatment | 4 | 0.4 | 2 | 0.5 | -50.0 |
| D3: Neuroleptics with moderate-marked antimuscarinic/anticholinergic effects with a history of prostatism or previous urinary retention | 1 | 0.1 | 1 | 0.3 | 0.0 |
| D4: Selective serotonin re-uptake inhibitors (SSRI's) with current or recent significant hyponatraemia i.e. serum Na+ < 130 mmol/l | 5 | 0.5 | 1 | 0.3 | -80.0 |
| D5: Benzodiazepines for ≥ 4 weeks | 223 | 20.7 | 82 | 20.8 | -63.2 |
| D7: Anticholinergics/antimuscarinics to treat extra-pyramidal side-effects of neuroleptic medications | 1 | 0.1 | 1 | 0.3 | 0.0 |
| D8: Anticholinergics/antimuscarinics in patients with delirium or dementia | 7 | 0.7 | 4 | 1.0 | -42.9 |
| D9: Neuroleptic antipsychotic in patients with behavioural and psychological symptoms of dementia (BPSD) unless symptoms are severe and other non-pharmacological treatments have failed | 2 | 0.2 | 1 | 0.3 | -50.0 |
| E1: Digoxin at a long-term dose greater than 125µg/day if eGFR < 30 ml/min/1.73m2 | 3 | 0.3 | 0 | 0 | -100 |
| E4: NSAID's if eGFR < 50 ml/min/1.73m2 | 9 | 0.8 | 4 | 1.0 | -55.6 |
| E6: Metformin if eGFR < 30 ml/min/1.73m2 | 8 | 0.7 | 0 | 0 | -100 |
| F2: PPI for uncomplicated peptic ulcer disease or erosive peptic oesophagitis at full therapeutic dosage for > 8 weeks | 7 | 0.7 | 3 | 0.8 | -57.1 |
| F3: Drugs likely to cause constipation in patients with chronic constipation where non-constipating alternatives are available | 1 | 0.1 | 1 | 0.3 | 0.0 |
| F4: Oral elemental iron doses greater than 200 mg daily | 2 | 0.2 | 1 | 0.3 | -50.0 |
| G2: Systemic corticosteroids instead of inhaled corticosteroids for maintenance therapy in moderate-severe COPD | 2 | 0.2 | 1 | 0.3 | -50.0 |
| G3: Anti-muscarinic bronchodilators (e.g. ipratropium, tiotropium) with a history of narrow angle glaucoma or bladder outflow obstruction | 2 | 0.2 | 3 | 0.8 | 50.0 |
| G4: Non-selective beta-blocker (whether oral or topical for glaucoma) with a history of asthma requiring treatment | 4 | 0.4 | 0 | 0 | -100 |
| G5: Benzodiazepines with acute or chronic respiratory failure i.e. pO2 < 8.0 kPa ± pCO2 > 6.5 kPa | 46 | 4.3 | 9 | 2.3 | -80.4 |
| H2: NSAID with severe hypertension or severe heart failure | 5 | 0.5 | 1 | 0.3 | -80.0 |
| H3: Long-term use of NSAID (>3 months) for symptom relief of osteoarthritis pain where paracetamol has not been tried | 1 | 0.1 | 0 | 0 | -100 |
| H5: Corticosteroids (other than periodic intra-articular injections for mono-articular pain) for osteoarthritis | 1 | 0.1 | 0 | 0 | -100 |
| H7: COX-2 selective NSAIDs with concurrent cardiovascular disease | 1 | 0.1 | 0 | 0 | -100 |
| I1: Antimuscarinic drugs with dementia, or chronic cognitive impairment or narrow-angle glaucoma, or chronic prostatism | 11 | 1.0 | 8 | 2.0 | -27.3 |
| I2: Selective alpha-1 selective alpha blockers in those with symptomatic orthostatic hypotension or micturition syncope | 1 | 0.1 | 1 | 0.3 | 0.0 |
| J1: Sulphonylureas with a long duration of action (e.g. glibenclamide, chlorpropamide, glimepiride) with type 2 diabetes mellitus | 4 | 0.4 | 0 | 0 | -100 |
| J3: Beta-blockers in diabetes mellitus with frequent hypoglycaemic episodes | 2 | 0.2 | 1 | 0.3 | -50.0 |
| K1: Benzodiazepines | 114 | 10.6 | 28 | 7.1 | -75.4 |
| K2: Neuroleptic drugs | 24 | 2.2 | 12 | 3.1 | -50.0 |
| K3: Vasodilator drugs with persistent postural hypotension i.e. recurrent drop in systolic blood pressure ≥ 20mmHg | 4 | 0.4 | 0 | 0 | -100 |
| K4: Hypnotic Z-drugs e.g. zopiclone, zolpidem, zaleplon | 10 | 0.9 | 3 | 0.8 | -70.0 |
| L1: Use of oral or transdermal strong opioids as first line therapy for mild pain | 15 | 1.4 | 8 | 2.0 | -46.7 |
| L2: Use of regular (as distinct from PRN) opioids without concomitant laxative | 33 | 3.1 | 13 | 3.3 | -60.6 |
| L3: Long-acting opioids without short-acting opioids for break-through pain | 3 | 0.3 | 3 | 0.8 | 0.0 |
| N1: Concomitant use of two or more drugs with antimuscarinic/anticholinergic properties | 2 | 0.2 | 0 | 0 | -100 |
| Total | 1077 | 100 | 394 | 100 | -63.4 |

**Supplementary Table S5. All START criteria identified at admission and discharge.**

| Criteria | Admission | | Discharge | | % of change |
| --- | --- | --- | --- | --- | --- |
|  | N | % | N | % |  |
| A1: Vitamin K antagonists or direct thrombin inhibitors or factor Xa inhibitors in the presence of chronic atrial fibrillation | 18 | 5.5 | 5 | 3.4 | -72.2 |
| A2: Aspirin (75 mg – 160 mg once daily) in the presence of chronic atrial fibrillation, where Vitamin K antagonists or direct thrombin inhibitors or factor Xa inhibitors are contraindicated | 2 | 0.6 | 0 | 0 | -100 |
| A3: Antiplatelet therapy (aspirin or clopidogrel or prasugrel or ticagrelor) with a documented history of coronary, cerebral or peripheral vascular disease | 8 | 2.4 | 3 | 2.0 | -62.5 |
| A4: Antihypertensive therapy where systolic blood pressure consistently > 160 mmHg and/or diastolic blood pressure consistently >90 mmHg; if systolic blood pressure > 140 mmHg and /or diastolic blood pressure > 90 mmHg, if diabetic | 8 | 2.4 | 1 | 0.7 | -87.5 |
| A5: Statin therapy with a documented history of coronary, cerebral or peripheral vascular disease, unless the patient's status is end-of-life or age is > 85 years | 3 | 0.9 | 4 | 2.7 | 33.3 |
| A6: Angiotensin Converting Enzyme (ACE) inhibitor with systolic heart failure and/or documented coronary artery disease | 35 | 10.6 | 15 | 10.1 | -57.1 |
| A7: Beta-blocker with ischaemic heart disease | 13 | 3.9 | 4 | 2.7 | -69.2 |
| A8: Appropriate beta-blocker (bisoprolol, nebivolol, metoprolol or carvedilol) with stable systolic heart failure | 37 | 11.2 | 11 | 7.4 | -70.3 |
| B1: Regular inhaled b2 agonist or antimuscarinic bronchodilator (e.g. ipratropium, tiotropium) for mild to moderate asthma or COPD | 2 | 0.6 | 1 | 0.7 | -50.0 |
| B2: Regular inhaled corticosteroid for moderate-severe asthma or COPD, where FEV1 <50% of predicted value and repeated exacerbations requiring treatment with oral corticosteroids | 3 | 0.9 | 2 | 1.4 | -33.3 |
| B3: Home continuous oxygen with documented chronic hypoxaemia (i.e. pO2 < 8.0 kPa or 60 mmHg or SaO2 < 89%) | 0 | 0 | 3 | 2.0 | Inf |
| C2: Non-TCA antidepressant drug in the presence of persistent major depressive symptoms | 14 | 4.2 | 1 | 0.7 | -92.9 |
| C3: Acetylcholinesterase inhibitor (e.g. donepezil, rivastigmine, galantamine) for mild-moderate Alzheimer's dementia or Lewy Body dementia (rivastigmine) | 8 | 2.4 | 6 | 4.0 | -25.0 |
| C5: Selective serotonin reuptake inhibitor (or SNRI or pregabalin if SSRI contraindicated) for persistent severe anxiety that interferes with independent functioning | 2 | 0.6 | 1 | 0.7 | -50.0 |
| D1: Proton Pump Inhibitor with severe gastro-oesophageal reflux disease or peptic stricture requiring dilatation | 5 | 1.5 | 2 | 1.4 | -60.0 |
| D2: Fibre supplements (e.g. bran, ispaghula, methylcellulose, sterculia) for diverticulosis with a history of constipation | 2 | 0.6 | 0 | 0 | -100 |
| E1: Disease-modifying anti-rheumatic drug (DMARD) with active, disabling rheumatoid disease | 1 | 0.3 | 1 | 0.7 | 0.0 |
| E2: Bisphosphonates and vitamin D and calcium in patients taking long-term systemic corticosteroid therapy | 10 | 3.0 | 11 | 7.4 | 10.0 |
| E3: Vitamin D and calcium supplement in patients with known osteoporosis and/or previous fragility fracture(s) and/or (Bone Mineral Density T-scores more than -2.5 in multiple sites) | 20 | 6.1 | 15 | 10.1 | -25.0 |
| E4: Bone anti-resorptive or anabolic therapy (e.g. bisphosphonate, strontium ranelate, teriparatide, denosumab) in patients with documented osteoporosis, where no pharmacological or clinical status contraindication exists (Bone Mineral Density T-scores -> 2.5 in multiple sites) and/or previous history of fragility fracture(s). | 7 | 2.1 | 4 | 2.7 | -42.9 |
| E5: Vitamin D supplement in older people who are housebound or experiencing falls or with osteopenia (Bone Mineral Density T-score is > -1.0 but < -2.5 in multiple sites) | 74 | 22.4 | 18 | 12.2 | -75.7 |
| E6: Xanthine-oxidase inhibitors (e.g. allopurinol, febuxostat) with a history of recurrent episodes of gout | 3 | 0.9 | 2 | 1.4 | -33.3 |
| F1: ACE inhibitor or Angiotensin Receptor Blocker (if intolerant of ACE inhibitor) in diabetes with evidence of renal disease i.e. dipstick proteinuria or microalbuminuria (>30mg/24 hours) with or without serum biochemical renal impairment. | 4 | 1.2 | 2 | 1.4 | -50.0 |
| G1: Alpha-1 receptor blocker with symptomatic prostatism, where prostatectomy is not considered necessary | 2 | 0.6 | 5 | 3.4 | 150.0 |
| G2: 5-alpha reductase inhibitor with symptomatic prostatism, where prostatectomy is not considered necessary | 4 | 1.2 | 5 | 3.4 | 25.0 |
| H1: High-potency opioids in moderate-severe pain, where paracetamol, NSAIDs or low-potency opioids are not appropriate to the pain severity or have been ineffective | 2 | 0.6 | 0 | 0 | -100 |
| H2: Laxatives in patients receiving opioids regularly | 43 | 13.0 | 26 | 17.6 | -39.5 |
| Total | 330 | 100 | 148 | 100 | -55.2 |

**Supplementary Figure legend**

**Figure S1. Clinical committee review process reduces the number of STOPP and START criteria at patients’ discharge. (A)** Distribution of patients according to the number of STOPP criteria identified at admission (green) and at discharge (purple). **(B)** Distribution of patients according to the number of START criteria identified at admission (green) and at discharge (purple). In both panels, percentages displayed are calculated on the total of patients at each time point.
